# Cardiac markers in left-sided breast cancer patients receiving adjuvant radiotherapy: a prospective study

**DOI:** 10.1186/s40959-024-00225-1

**Published:** 2024-04-08

**Authors:** Kundan Chufal, Irfan Ahmad, Anuj Prakash, Alexis Miller, Preetha Umesh, Varsha Koul, Ram Bajpai, Bharat Dua, Priya Gupta, Munish Gairola

**Affiliations:** 1 Department of Radiation Oncology, Rajiv Gandhi Cancer Institute and Research Centre, New Delhi, India; 2 Department of Biochemistry, Medanta Hospital, Gurugram, Haryana India; 3 Department of Radiation Oncology, Illawara Cancer Care Centre, Wollongong, NSW Australia; 4 Department of Cardiology, Batra Hospital and Medical Research Centre, New Delhi, India; 5 School of Medicine, Keele University, Staffordshire, UK; 6 Department of Radiation Oncology, Venkateshwar Hospital, New Delhi, India

**Keywords:** Breast cancer, hsTnI, Hypofractionated Radiotherapy, NT-proBNP, hsCRP

## Abstract

**Objectives:**

To investigate the association between radiotherapy (RT) and cardiac biomarkers in women with left-sided breast cancer.

**Methods:**

This prospective observational study recruited patients with stage I-III left-sided breast cancer without coronary heart disease who required adjuvant RT. High-sensitivity troponin I(hsTnI), N-terminal pro-brain natriuretic peptide(NT-proBNP), and high-sensitivity C-reactive protein(hsCRP) levels were measured pre-RT, immediately after RT, and 3 months post-RT. Cardiac-sparing RT techniques were utilized (Field-in-Field IMRT/VMAT ± voluntary deep inspiration breath-hold). Statistical analyses were performed using non-parametric tests and multivariable quantile regression (QR).

**Results:**

One hundred five patients completed the study, with 63 evaluable at three months post-RT. Pre- and post-RT biomarkers showed no significant differences. Median pre-RT and post-RT values were: hsTnI (0.012ng/mL; 0.012ng/mL), hsCRP (3.1 mg/L; 2.8 mg/L), and NT-proBNP (59pg/mL; 45pg/mL). Three months post-RT, hsTnI, hsCRP and NT-proBNP levels also showed no significant differences. Multivariable QR revealed no association between heart D_mean_ [median(IQR): 2.87 Gy (2.05–3.94)] and post-RT biomarkers. Age and BMI were associated with hsCRP and NT-proBNP, respectively.

**Conclusions:**

hsTnI, NT-proBNP, and hsCRP are not correlated with contemporary low cardiac exposure in left-sided breast cancer patients treated with contemporary RT techniques.

**Supplementary Information:**

The online version contains supplementary material available at 10.1186/s40959-024-00225-1.

## Background

Cardiovascular diseases (CVD) in cancer patients are driven by treatment-related risk, which spans therapeutic classes and shares damage mechanisms leading to combined toxicity [1–3]. Cardio-oncology guidelines endorse surveillance with blood-based biomarkers (troponins and natriuretic peptides) and cardiac imaging [4]. Troponins are cardiac-specific, but not disease-specific, and natriuretic peptides are associated with heart failure (HF) [5]. Oxidative stress and inflammation induce C-reactive protein secretion, which is associated with poor outcomes in decompensated HF [5]. Association between radiotherapy (RT) and these biomarkers is inconclusive, therefore specific recommendations for radiation-induced cardiac damage are absent [4]. 

Our primary objectives were to evaluate: (a) the effect of RT on biomarkers reflective of myocardial injury/inflammation [high-sensitivity cardiac troponin I (hsTnI), N-terminal pro brain natriuretic peptide (NT-proBNP) and oxidative stress [high-sensitivity C-reactive protein (hsCRP)], and; (b) the association between mean heart dose (D_mean_) and biomarkers.

## Methods

### Study population

This prospective, single-institution, IRB-approved (Protocol ID: Res/SCM/52/2022/40; IRB Approval ID: IRB-BHR/75/2022) observational study was conducted between June 2022 and July 2023. Women with left-sided breast cancer were eligible. Inclusion criteria were: (a) greater than 18 years with pathological stage I-III disease after either breast conservation surgery or mastectomy; (b) without coronary heart disease (CHD) or CHD risk equivalent; (c) requiring adjuvant RT (42.5 Gy/16Fx, 5 days/week) to whole breast or chest wall with/without elective regional nodal irradiation (sequential lumpectomy boost permitted; 10 Gy in 4Fx), and; (d) normal 2D transthoracic echocardiography prior to starting RT. Chemotherapy (neoadjuvant/adjuvant)(with trastuzumab for Her2-expressing tumors) was permitted. Adjuvant RT was delivered 3–4 weeks after completion of the preceding surgery or chemotherapy.

### Study procedures

After obtaining informed consent, demographic, clinical, CVD risk factors, and treatment details were collected. Blood samples were collected pre-RT, immediately after RT completion (median: 0 days, Range: 0–18 days), and three months after RT completion (median: 92 days, IQR: 90–99 days). Since only 30% of patients return for a 3-month review post-RT (internal audit), 3-month biomarker measurement was optional. After accruing 52 patients, the data monitoring committee excluded hsTnI from the panel of tests, as 40 patients had values below the detection limit (0.012 ng/ml) at pre- and post-RT time points.

All patients were assessed for treatment in deep inspiration breath hold (DIBH)(RPM system, Varian Medical Systems, USA) and received RT via Field-in-Field Intensity-Modulated Radiotherapy Technique (FiF IMRT) or Volumetric Modulated Arc Therapy on a 6MV LINAC with daily kV-MV verification (Clinac 2100c, Varian Medical Systems, USA) [6]. Contouring for primary, nodal regions and organs-at-risk was performed as per RTOG 0413 protocol, RTOG consensus recommendations, and RTOG 1005 protocol (NCT01349322), respectively [7, 8]. Mean heart dose (D_mean_) was recorded for the whole heart contour using a calculation grid size of 2.5 mm with AAA v15.6 algorithm (Varian Medical Systems, USA).

### Biomarker measurements

All assays were performed on the Vitros 5600 platform (QuidelOrtho, USA). The hsCRP assay had a detection limit of 0.26 mg/L, coefficient of variation (CV) of < 8.3% at the 99th percentile with a reference limit of < 5.0 mg/L. The NT-proBNP assay had a measurement range of 11.1–35,000 pg/mL, CV of 11% at the 99th percentile with a reference limit of 125 pg/mL and 450 pg/mL for patients less than or greater than 75 years, respectively. The hsTnI assay had a measurement range of 0.012–80.0 ng/ml, CV of < 8.0% at the 99th percentile with a reference limit of 0.034 ng/mL. Whenever an elevated biomarker was detected, a cardiologist obtained a cardiac history and performed a cardiac examination.

### Statistical analysis

The sample size for a relative effect size of 30% with 80% power and alpha = 0.05 with a two-tailed, paired-sample design assuming a normal distribution was 94. Another 15% were added for dropout, resulting in a sample size of 108. Baseline characteristics were reported as median with interquartile range (IQR)(continuous variables) or frequencies and percentages (categorical variables). All biomarkers had non-normal distribution; therefore, the Wilcoxon matched-pairs signed-rank test was used to compare pre- and post-RT levels with tied pairs handled by Pratt’s method [9]. The Friedman test was used to compare biomarkers at pre-, post-RT, and 3-months post-RT. Paired comparisons between time points utilized Dunn’s post-test [10]. Spearman rank correlation was used to assess correlation between biomarkers.

Multivariable quantile regression (QR) was used to test association of biomarkers with predictors without considering interactions [11]. Compared to ordinary least-squares (OLS) regression, QR demonstrates robust performance in non-normal distributions and in the presence of outliers. The Markov chain marginal bootstrap determined the standard error [12]. QR results were compared to OLS regression after log-transformation of biomarkers (Supplemental Materials).

This report complies with the ‘Strengthening the Reporting of Observational Studies in Epidemiology cohort’ guidelines (Supplemental Materials). Statistical analyses were performed using Prism v10 (DotMatics, USA) and R v4.2.3 (R Foundation for Statistical Computing, Austria). Statistical significance was set at *p* < 0.05 (two-sided).

## Results

**Fig. 1:**
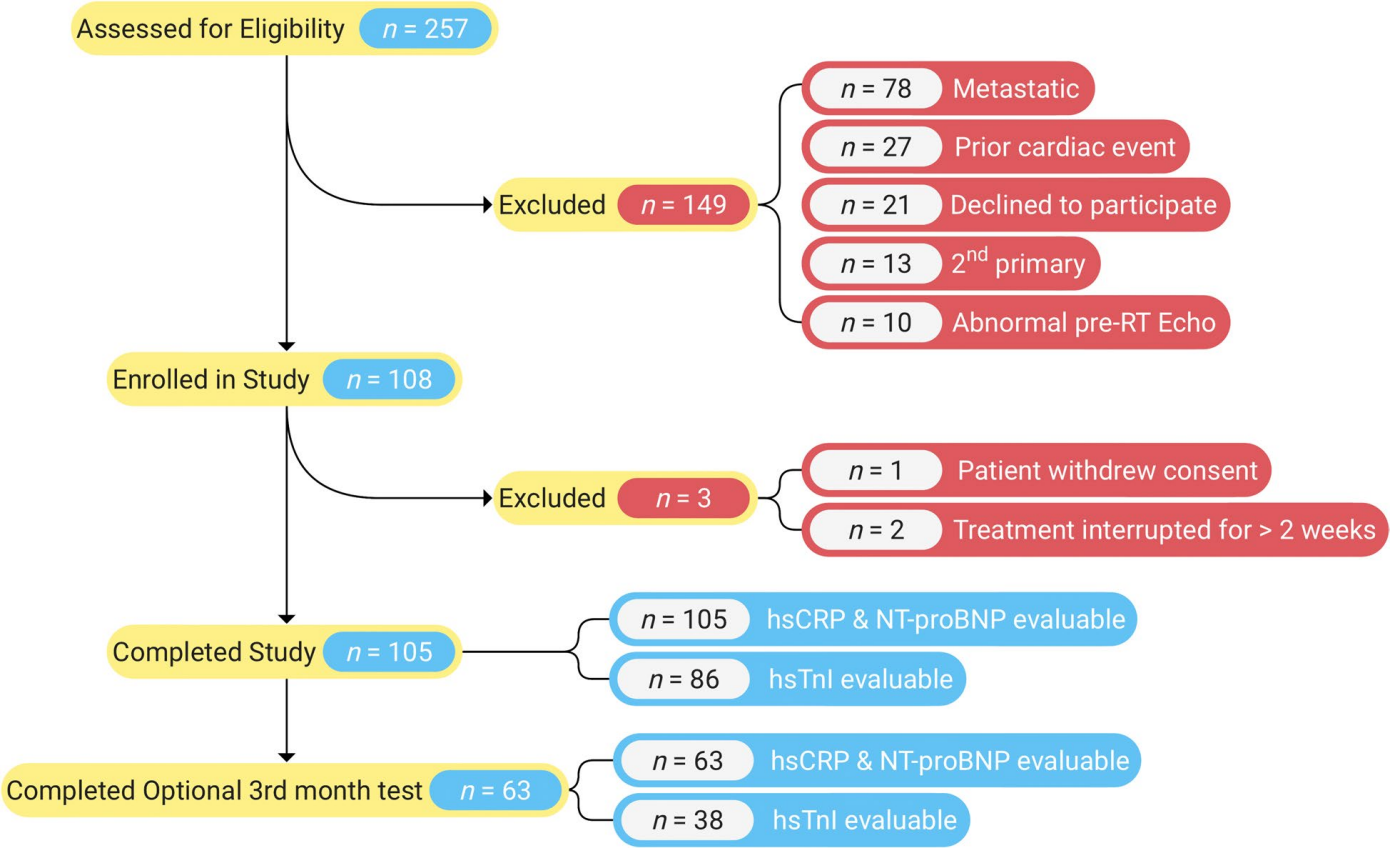
Study flow diagram

One hundred five patients completed the study and were analyzed for pre-/post-RT comparison, while 63 patients completed the 3-months post-RT evaluation (Fig. 1). Because the data monitoring committee recommended stopping hsTnI testing, 86 patients were analyzable for pre-/post-RT comparison, and 38 were analyzable at all three time points. The patients’ baseline characteristics are presented in Table 1. There was no correlation between markers (Supplemental materials). None of the patients developed a Major Adverse Cardiac Event (MACE) with a median follow-up of 15.0 months (IQR: 12.9–17.3) after the completion of RT. In addition, all patients with elevated biomarkers were asymptomatic on specialist examination, required no additional investigations and completed treatment without interruptions (Supplemental Materials).

**Table 1:** Baseline Characteristics (*n* = 105) of the study population

	n	%
Median Age - years (IQR)	51 (45–58)	-
BMI (Kg/m^2^) (IQR)	28.2 (24.5–30.8)	
Current Smoking	2	2%
Hypertension	28	27%
Surgery performed		
Breast conservation surgery	57	54%
Modified radical mastectomy	48	46%
Type of systemic treatment received		
Anthracycline based	62	59%
Anti-Her2 based	22	21%
Without anthracycline or anti-Her2	19	18%
Both anthracyclines and anti-Her2	2	2%
Radiotherapy details		
DIBH / Non-DIBH	51 / 54	49% / 51%
FiF IMRT / VMAT	87 / 18	83% / 17%
Elective nodal regions irradiated	89	85%
Median whole heart D_mean_ (Gy) (IQR)	2.87 (2.05–3.94)	-

*Abbreviations*: *AJCC *American Joint Committee on Cancer, *BMI *Body Mass Index, *DIBH *Deep Inspiration Breath Hold; D_mean_, Mean Dose, *Ed *Edition, *FiF IMRT *Field-in-Field Intensity Modulated Radiotherapy Technique, *Gy *Gray, *IQR *Inter-Quartile Range, *VMAT *Volumetric Modulated Arc Therapy

### Biomarker comparison: Pre- vs. Post-RT (Fig. 2)

**Fig. 2:**
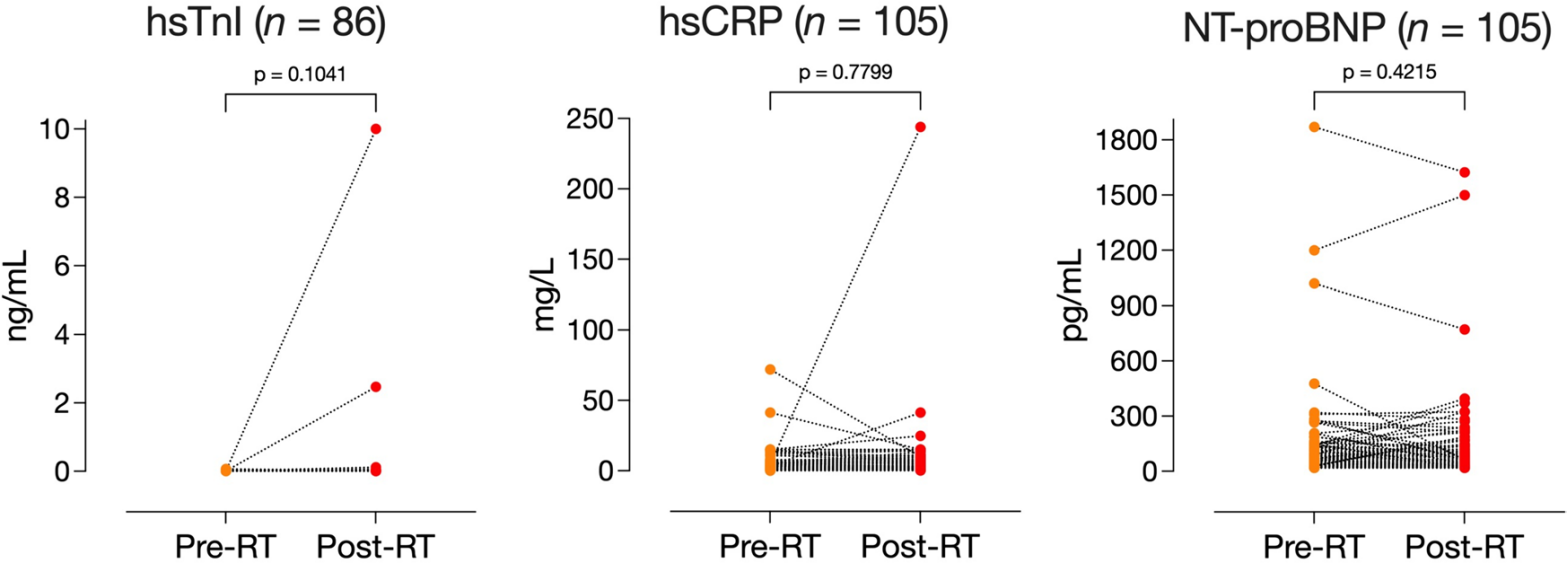
Wilcoxon signed-rank test, comparing biomarkers at pre- and post-RT time points

The median (IQR) values of hsTnI, hsCRP, and NT-proBNP pre-RT were 0.012 ng/mL (0.012–0.012), 3.1 mg/L (1.5-6.0) and 59 pg/mL (26–116), respectively. The post-RT biomarkers were 0.012 ng/mL (0.012–0.012), 2.8 mg/L (1.6–6.8), and 45 pg/mL (24–101), respectively. The pre-/post-RT comparisons were not significantly different.

### Biomarker comparison: Pre-, Post- and 3 months after RT (Fig. 3)

**Fig. 3:**
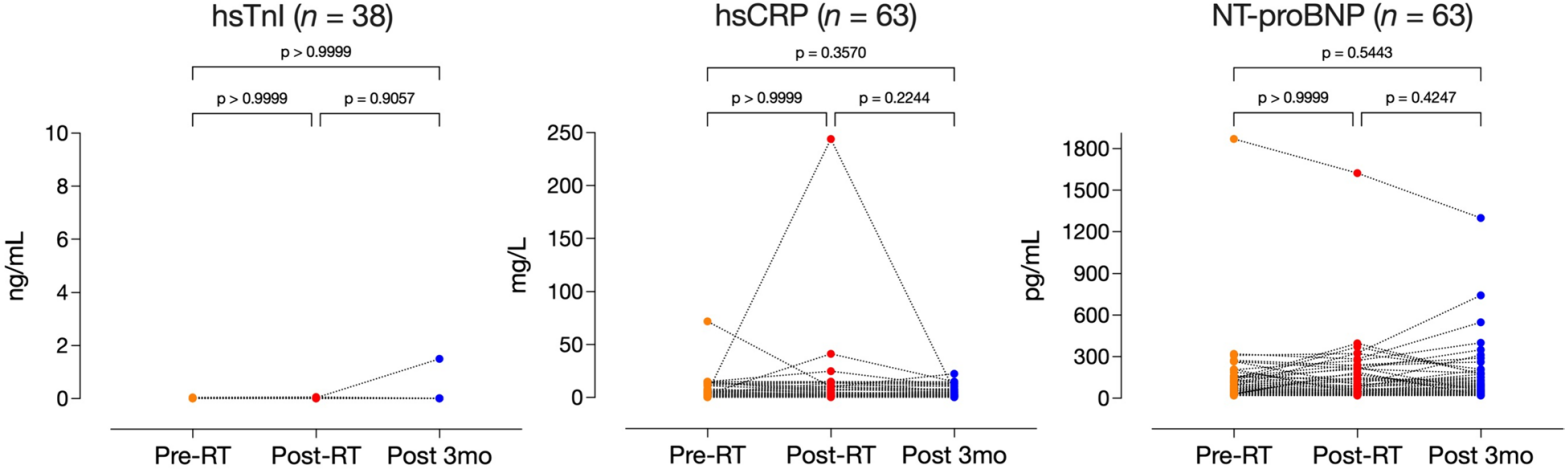
Friedman test, comparing biomarkers at pre-, post-, and 3-months post-RT time points

The median (IQR) values of hsTnI, hsCRP, and NT-proBNP pre-RT were 0.012 ng/mL (0.012–0.012), 3.1 mg/L (1.4–6.1) and 56 pg/mL (29–125), respectively. The post-RT levels were 0.012 ng/mL (0.012–0.012), 2.4 mg/L (1.5–6.8), and 50 pg/mL (28–143), respectively. 3-months post-RT, the levels were 0.012 ng/mL (0.012–0.012), 2.7 mg/L (1.1-6.0), and 60 pg/mL (31–151), respectively. Comparisons between pre-, post-, and 3-months post-RT values were not significantly different.

### Multivariable median quantile regression: pre- and Post-RT (Table 2)

**Table 2:** Results of multi-variable median quantile regression

	Pre-Radiotherapy				Post-Radiotherapy			
	hsCRP		NT-proBNP		hsCRP		NT-proBNP	
Intercept	-2.46 (3.48)	0.482	− 66.31 (62.88)	0.294	− 4.55 (3.22)	0.160	− 10.42 (48)	0.829
Age	− 0.04 (0.04)	0.323	2.32 (0.99)	**0.021**	− 0.07 (0.04)	0.072	1.50 (0.75)	**0.048**
BMI	0.26 (0.10)	**0.013**	− 0.12 (2.09)	0.955	0.39 (0.10)	**< 0.001**	− 0.24 (1.48)	0.869
Hypertensive (vs. no)	0.49 (0.99)	0.620	5.80 (18.75)	0.758	2.07 (1.31)	0.117	20.56 (22.99)	0.373
Received Anthracyclines (vs. no)	0.87 (1.09)	0.425	12.98 (32.30)	0.689	− 0.75 (0.83)	0.370	16.41 (14.30)	0.254
Received Anti-Her2 (vs. no)	0.75 (1.36)	0.580	− 0.88 (35.88)	0.980	− 1.03 (1.04)	0.327	− 9.82 (14.51)	0.500
Whole heart D_mean_	NM	-	NM	-	0.49 (0.28)	0.081	− 6.11 (4.06)	0.13
	Estimate (SE)	*p*	Estimate (SE)	*p*	Estimate (SE)	*p*	Estimate (SE)	*p*

*Abbreviations*: *BMI* Body Mass Index, *D*_*mean*_ Mean Dose, *NM* Not Modelled

Multivariable median quantile regression demonstrated no influence of heart D_mean_ on any post-RT biomarker. No treatment-related factors (anthracycline or anti-Her2 exposure) influenced pre- or post-RT biomarkers. BMI and age were related to hsCRP and NT-proBNP at pre- and post-RT measurements, respectively. hsTnI analysis was not clinically meaningful as the variables had an estimate of < 0.0001 (Supplemental Materials), because the measurements were below the detection threshold. Finally, the results of QR were comparable to OLS regression (Supplemental materials).

## Discussion

Identifying measurable markers associated with cardiac radiation exposure may permit MACE risk stratification of patients during follow-up and provide an objective measure to stratify risks with emerging RT techniques. Despite two decades of research, a reliable association has not been established (Table 3) [13–26]. Therefore, this prospective study of predefined, serial time-point biomarker measurements was deliberately designed in a homogeneous low-risk population of patients with left-sided breast cancer who received standardized modern RT to reduce variability, thereby isolating and enhancing the probability of detecting *any* RT effect. The biomarkers were chosen based on their role in monitoring cardiotoxic therapies and availability in community practice, in contrast to candidate research markers, which require specialized/centralized testing laboratories [25, 26].

**Table 3:** Overview of the literature on the association between cardiac biomarkers and cardiac radiation exposure. (Note: The criteria for inclusion were: (a) patients with breast cancer had to be treated with radiotherapy, and; (b) any cardiac biomarker was tested to determine its association with radiotherapy. Studies which measured biomarker change in relation to chemotherapy alone or were performed in animals, were excluded.)

Author, year [Ref]	Design	*n*	Population	RT dose^a^	Technique^a^	Anthracycline/ Anti-Her2 therapy	Mean (SD) Heart D_mean_	Biomarkers measured	Biomarker measurement timepoints	Reported findings on the association of biomarkers with cardiac dosimetry	Comments^c^
Studies without reported Heart D_mean_											
Hughes-Davies et al., 1995 [13]	Prospective	50	Left Breast cancer	45-46 Gy/23-25Fx + boost	Conventional Photon	Not Available	NR	TnT	Pre-RT and immediately post-RT	No association of TnT with estimated cardiac irradiation (< 10%)	Indirect estimation of irradiated cardiac volume
Wondergem et al., 2001 [14]	Case–control; retrospective case selection	188	Breast cancer (48; L/R = NR), Hodgkin’s lymphoma (73) *vs* Healthy Controls (67)	50-60 Gy/20-30Fx	Conventional Photon	Not Available	NR^b^	ANP	9.5 years (mean; SD = 0.9) post-RT	ANP elevated with estimated cardiac irradiation (20–30%) compared to control group. Elevated ANP associated with CVD	Multi-variable analysis of predictors for ANP not performed. Indirect estimation of irradiated cardiac volume
D’Errico et al., 2015 [18]	Prospective	59	Left breast cancer	40-50 Gy/15-25Fx	3DCRT	Permitted	NR^d^	TnI, BNP	Pre-RT, during RT, immediately post-RT and 1, 3, 6, 9, 12 months post-RT	Heart V50% was associated with normalised BNP at 1 year. ^e^ No association with TnI	Logistic regression performed after dosimetric variables and BNP at 1 year were categorised (full model NR). Analysis of absolute measurements not performed. No correction for multiple comparisons
Chalubinska-Fendler et al., 2019 [23]	Case–control; Prospective	51	Left breast cancer	50-66 Gy/25-33Fx	3DCRT	Permitted	NR	LBP, TnT, NT-proBNP, FABP, CRP	Pre-RT, immediately after RT and 1 month after RT	LBP levels were associated with cardiac dosimetry on multivariable linear regression. No association of other biomarkers with cardiac dosimetry	-
Aula et al., 2020 [25]	Prospective	63	Breast cancer (L/R = 50/13)	50 Gy/25Fx ± boost *or* 42.6 Gy/16Fx	3DCRT	Not Permitted	NR^b^	sST2,	Pre-RT, immediately after RT and 3 months after RT	Patients with > 15% worsening in global longitudinal strain (GLS)(14/63) on echocardiography showed a significant increase in ST2 levels. No association of biomarkers with cardiac dosimetry	Logistic regression performed after patients were categorised based on GLS cut-off of 15%. Analysis of absolute measurements with dosimetric variables not performed
Studies with reported Heart D_mean_											
Erven et al., 2012 [15]	Prospective	75	Breast cancer (L/R = 51/24)	50 Gy/25Fx ± boost	3DCRT, mixed photon-electron or electrons	Permitted	9.0 (4.0)^b^	TnI	Pre-RT and immediately post-RT	TnI levels were significantly elevated post-RT in left-sided patients	Multi-variable analysis of predictors for TnI not performed
D’Errico et al., 2012 [16]	Case–control; retrospective case selection	60	Left breast cancer (30, Pre-RT *vs* 30, Post RT)	40-50 Gy/15-25Fx + boost	3DCRT	Permitted	2.5 (1.2)	TnI, NT-proBNP	11.2 months (mean; SD = 4.2) post-RT	No correlation of cardiac dosimetry with overall post-RT NT-proBNP or TnI	Small sample size. Association of post-RT NT-proBNP with cardiac dosimetry was established on a small subgroup (8/30) of patients
Skyttä et al., 2015 [17]	Prospective	58	Left breast cancer or DCIS	50 Gy/25Fx ± boost *or* 42.6 Gy/16Fx	3DCRT	Not Permitted	3.0 (1.4)	hsTnT, BNP	Pre-RT, during RT and immediately post-RT	Patients with hsTnT rise > 30% (12/58) had higher heart D_mean_. No association with BNP	Exclusion of chemotherapy limits generalisability
Palumbo et al., 2015 [19]	Prospective	43	Left breast cancer	50–50.4 Gy/25-28Fx ± boost	3DCRT	Not Permitted	2.4 (0.8)	BNP	Pre-RT and 1, 6, 12 months post-RT	Normalised BNP at 1 month,, 6 months and 1 year post-RT were associated with cardiac dosimetry. ^e^ No association with absolute BNP levels	Small sample size. Exclusion of chemotherapy limits generalisability
Skyttä et al., 2019 [20]	Prospective	80	Breast cancer or DCIS (L/R = 60/20)	50 Gy/25Fx ± boost *or* 42.6 Gy/16Fx	3DCRT	Not Permitted	3.1 (1.5)^b^	hsTnT, NT-proBNP	Pre-RT, immediately after RT and 3 years after RT	No association of biomarkers with cardiac dosimetry	Exclusion of chemotherapy limits generalisability
Demissei et al., 2019 [21]	Prospective	87	Breast cancer (60; L/R = NR), Lung Cancer (13), Mediastinal lymphoma (14)	Conventional fractionation)	Photon (technique NR) or Protons	Permitted	1.5 (1.1)^b^	hsTnT, NT-proBNP, PIGF, GDF-15	Pre-RT and a median of 20 days post-RT (IQR 1–35)	No association of biomarkers with cardiac dosimetry in breast cancer patients	PIGF and GDF-15 associated with cardiac dosimetry in lung and lymphoma group, though sample size was small. Analysis for left breast not performed
Yu et al., 2019 [22]	Retrospective	47	Her2 + breast cancer (L/R = 26/21)	50 Gy/25Fx *or* 42.4 Gy/16Fx ± boost	3DCRT or IMRT	Permitted	1.8 (1.5)^b^	hsTnI	Baseline (Pre-Chemotherapy), Pre-RT, immediately post-RT and 6 months post-RT	Statistical analysis not performed due to incomplete data on paired samples	-
De Sanctis et al., 2020 [24]	Prospective	44	Breast cancer (L/R = 27/17)	42.4 Gy/16Fx ± boost	3DCRT	Permitted	1.3 (NR)^b^	hsTnI, NT-proBNP	Pre-RT, during RT, immediately post-RT and 12 months post-RT	No association of biomarkers with cardiac dosimetry	Heart D_mean_ not used for linear mixed modelling. Small sample size
Speers et al., 2021 [26]	Prospective	51	Left breast cancer	50 Gy/25Fx ± boost	3DCRT or IMRT	Permitted	2.0 (NR)	hsTnI, NT-proBNP, hsCRP, ET-1, IL-6, Lipid Profile ^f^	Pre-RT, immediately after RT and 3 months after RT	IL-6 immediately after completing RT, associated with heart D_mean_. No association of other biomarkers with cardiac dosimetry	Multi-variable analyses of predictors for biomarkers not performed

^a^In studies with different populations, RT dose and technique for only breast cancer patients are shown here

^b^In studies which included both left- and right-sided breast cancer patients or included different populations, the reported mean of only left-sided patients are shown here

^c^None of the studies performed a power and sample size calculation, except Chalubinska-Fendler et al., 2019

^d^Heart Dmean for the population was split into tertiles and reported

^e^ Normalised refers to converting the absolute value at a time point to a ratio, by dividing it by the baseline value

^f^ Lipid profile included cholesterol, triglycerides, high-density lipoproteins and low-density lipoproteins

*Abbreviations*: *ANP *Atrial Natriuretic Peptide, *3DCRT *3-Dimensional Conformal Radiotherapy, *BNP *Brain Natriuretic Peptide, CRP C-Reactive Protein, *CVD *Cardio-Vascular Disease, *DCIS *Ductal Carcinoma In-Situ, *ET-1* Endothelin-1, *FABP *Fatty Acid Binding Protein, *Fx *Fractions, *GDF-15 *Growth Differentiation Factor 15, *Gy *Gray, *hsTnI* High Sensitivity Troponin I, *hsTnT *High Sensitivity Troponin T, *IL-6 *Interleukin-6, *IMRT *Intensity Modulated Radiotherapy Technique, *IQR *Inter-Quartile Range, *LBP *Lipopolysaccharide-Binding Protein,  *L/R *Left/Right, *NR *Not Reported, *NT-proBNP *N-terminal pro brain natriuretic peptide, *PIGF *Placental Growth Factor, *RT *Radiotherapy, *SD *Standard Deviation, *sST2 *soluble Suppression of Tumorigenicity 2, *TnI *Troponin I, *TnT *Troponin T

Early investigations reported elevation in troponins and natriuretic peptides with RT but were limited by retrospective design or indirect estimates of irradiated heart volume [13, 14]. Subsequent analyses were limited by modest sample sizes and statistical power, precluding conclusive association between RT and biomarkers [15, 17–19, 26]. In contrast, this study was adequately powered to detect a 30% change in biomarkers and used rigorous QR and OLS regression strategies with consistent results.

The achieved median heart D_mean_ (2.87 Gy) demonstrates that exposure can be minimized in the real world, and the threshold for measurable cardiac damage using these biomarkers is clearly above this dose. This is supported by the fact that the majority of reported heart D_mean_ of individual studies was around 3 Gy (Table 3), without clear association with absolute cardiac biomarker change. Contemporaneous cardiac damage detection will require ultrasensitive biomarkers or long-term studies of cardiac outcomes to establish the magnitude of effect (NCT04361240; NCT04790266; NCT03297346). However, it must be emphasized that the reducing cardiac exposure is adequately achieved with FiF IMRT, which was developed two decades ago and remains the benchmark for comparing newer, more complicated RT techniques [27]. 

The sample size was designed to be adequate to detect changes in biomarkers, but we acknowledge that it is modest for detecting subtler temporal trends. We intend to follow this cohort biennially for 15 years to record MACE, and perform a post-hoc analysis in the future, if statistically appropriate. We also did not measure these biomarkers pre-chemotherapy because we intended to establish association with RT [4]. Since heart D_mean_ is the most validated dosimetric parameter for cardiac outcomes, we chose not to investigate association with other sub-structures or parameters thereof, to avoid creating a multiple testing problem [28]. Enrolling right-sided breast cancer patients as controls was considered. But since their risk of developing RT-induced CVD is extremely low, repeated blood investigations were deemed unwarranted by our IRB. Our study population was intentionally composed of patients with low cardiac risk, to isolate the effect of radiotherapy on cardiac markers. It is plausible that patients at higher cardiac risk could demonstrate a more pronounced change in cardiac markers in response to radiotherapy, and could be an avenue for future research.

In conclusion, the lack of correlation between these biomarkers and cardiac radiation exposure will aid in narrowing the scope of future research. These results and prior reports clearly argue *against* their routine use to detect radiotherapy-induced cardiac injury with modern RT techniques.

### Supplementary Information

**Supplementary Material 1.**

## Data Availability

This study was performed at Rajiv Gandhi Cancer Institute & Research Centre and is stored in the institutions data repository. The authors do not own these data and hence are not permitted to share them in the original form (only in aggregate form). Reasonable requests for access to data will be considered on an individual basis, by contacting the corresponding author.
