# Supplementary material for: Cardiac markers in left-sided breast cancer patients receiving adjuvant radiotherapy: a prospective study
Source: Cardiooncology. 2024 Apr 8;10:21. doi: 10.1186/s40959-024-00225-1 (PMC11000277; doi:10.1186/s40959-024-00225-1)
Supplement: Supplementary file 1 — Supplementary Material 1. [file 40959_2024_225_MOESM1_ESM.docx]

|  | Pre-Radiotherapy | | Post-Radiotherapy | |
| --- | --- | --- | --- | --- |
|  | hsTnI | | hsTnI | |
|  | Estimate (SE) | *p* | Estimate (SE) | *p* |
| Intercept | 0.01 (0.00) | **< 0.001** | 0.01 (0.01) | 0.111 |
| Age | 0.00 (0.00) | 0.235 | 0.00 (0.00) | 1.000 |
| BMI | 0.00 (0.00) | 0.936 | 0.00 (0.00) | 1.000 |
| Hypertensive (*vs* no) | 0.00 (0.00) | 0.249 | 0.00 (0.00) | 1.000 |
| Received Anthracyclines (*vs* no) | 0.00 (0.00) | 0.256 | 0.00 (0.00) | 1.000 |
| Received Anti-Her2 (*vs* no) | 0.00 (0.00) | 0.659 | 0.00 (0.00) | 1.000 |
| Whole heart D_mean_ | NM | - | 0.00 (0.00) | 1.000 |
| *Abbreviations*: BMI, Body Mass Index; D_mean_, Mean Dose; NM, Not Modelled | | | | |

Supplementary Table 1: Multivariable quantile regression for high sensitivity Troponin I.

|  | Pre-Radiotherapy | | | | | | Post-Radiotherapy | | | | | |
| --- | --- | --- | --- | --- | --- | --- | --- | --- | --- | --- | --- | --- |
|  | hsCRP | | NT-proBNP | | hsTnI | | hsCRP | | NT-proBNP | | hsTnI | |
|  | Estimate (SE) | *p* | Estimate (SE) | *p* | Estimate (SE) | *p* | Estimate (SE) | *p* | Estimate (SE) | *p* | Estimate (SE) | *p* |
| Intercept | -0.87 (0.36) | **0.017** | - 0.91 (0.31) | **0.004** | - 1.90 (0.11) | **< 0.001** | - 0.99 (0.32) | **0.003** | 1.25 (0.32) | **< 0.001** | - 1.80 (0.38) | **< 0.001** |
| Age | - 0.01 (0.01) | 0.697 | 0.02 (0.01) | **< 0.001** | - 0.00 (0.00) | 0.811 | - 0.01 (0.01) | 0.301 | 0.01 (0.01) | **0.001** | 0.00 (0.00) | 0.435 |
| BMI | 0.04 (0.01) | **< 0.001** | 0.01 (0.01) | 0.628 | - 0.01 (0.00) | 0.973 | 0.05 (0.01) | **< 0.001** | - 0.01 (0.01) | 0.719 | 0.00 (0.01) | 0.987 |
| Hypertensive (*vs* no) | - 0.11 (0.11) | 0.307 | - 0.02 (0.09) | 0.841 | 0.01 (0.03) | 0.696 | 0.14 (0.10) | 0.155 | 0.11 (0.10) | 0.282 | - 0.09 (0.11) | 0.380 |
| Received Anthracyclines (*vs* no) | 0.18 (0.12) | 0.132 | - 0.05 (0.10) | 0.602 | 0.05 (0.04) | 0.136 | 0.04 (0.11) | 0.718 | 0.02 (0.11) | 0.833 | - 0.23 (0.13) | 0.069 |
| Received Anti-Her2 (*vs* no) | 0.12 (0.13) | 0.355 | - 0.08 (0.17) | 0.464 | - 0.01 (0.04) | 0.944 | - 0.07 (0.12) | 0.532 | - 0.11 (0.12) | 0.376 | - 0.28 (0.15) | 0.055 |
| Whole heart D_mean_ | NM | - | NM | - | NM | - | 0.04 (0.03) | 0.190 | - 0.04 (0.03) | 0.112 | 0.01 (0.15) | 0.751 |
| *Abbreviations*: BMI, Body Mass Index; D_mean_, Mean Dose; NM, Not Modelled | | | | | | | | | | | | |

Supplementary Table 2: Results of Ordinary Lease Squares Regression modelling.


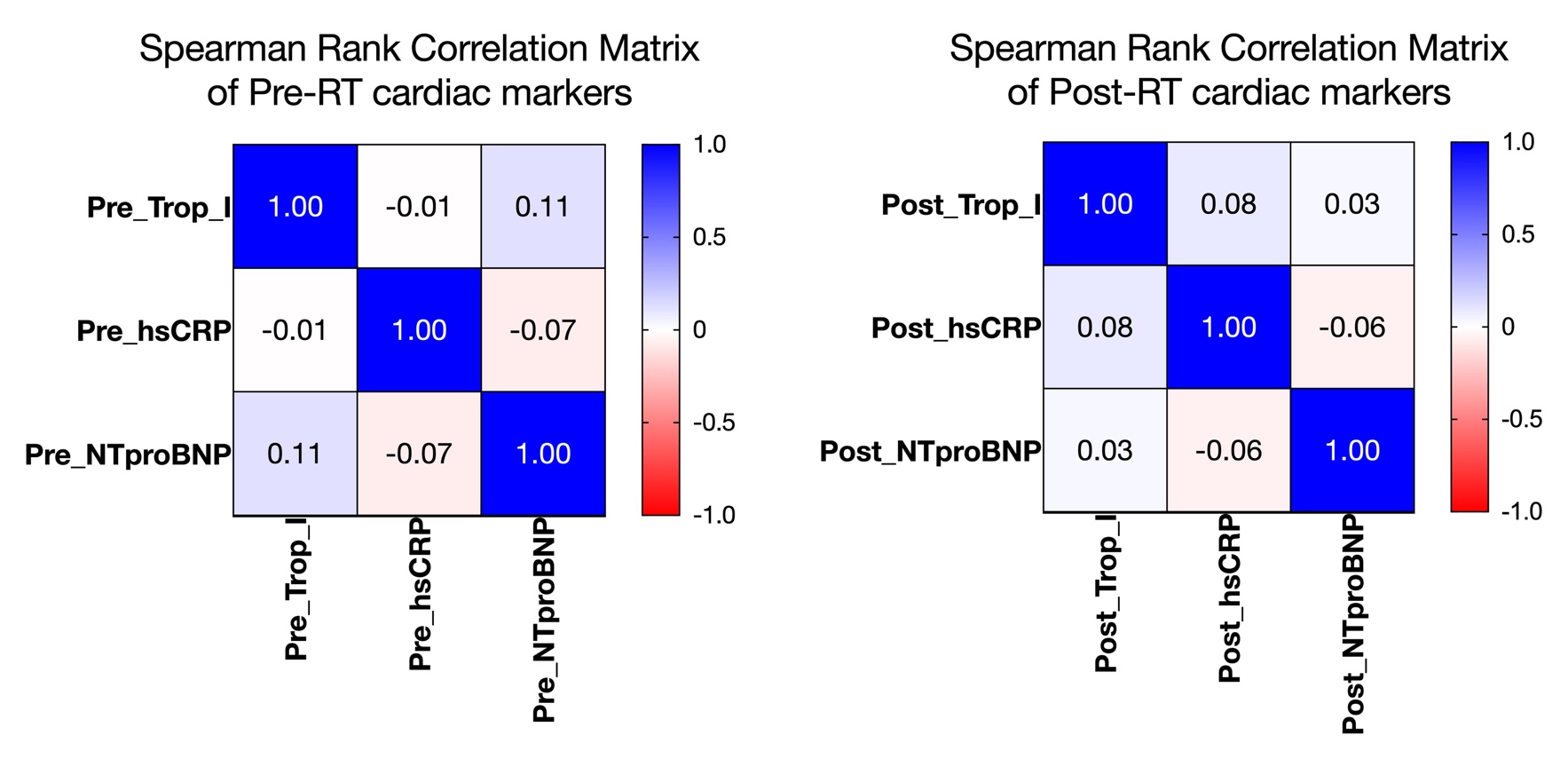


Supplementary Figure 1: Spearman rank correlation of Pre- and Post-RT biomarkers.


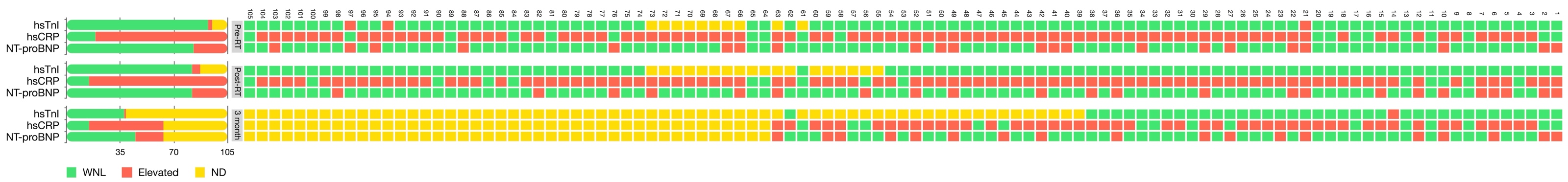


Supplementary Figure 2: Proportion of patients at each time point with normal or elevated biomarkers as a whole (Left). Individual patient data at each time-point (Right). Abbreviations: ND, Not Done; WNL, Within Normal Limits.
